# Native Aortic Valve Endocarditis Caused by *Scopulariopsis* Species: Case Report, Considerations for Management, and Review of Literature

**DOI:** 10.1093/ofid/ofae323

**Published:** 2024-06-14

**Authors:** Sabrina Imam, Christopher J Kaperak, Ahmed E Hozain, Hecong Qin, Cynthia T Nguyen, Praveen Sudhindra, Valluvan Jeevanandam, Emily Landon

**Affiliations:** Section of Infectious Diseases and Global Health, University of Chicago Medicine, Chicago, Illinois, USA; Section of Infectious Diseases and Global Health, University of Chicago Medicine, Chicago, Illinois, USA; Section of Cardiac Surgery, Department of Surgery, University of Chicago Medicine, Chicago, Illinois, USA; Pritzker School of Medicine, The University of Chicago, Chicago, Illinois, USA; Department of Pharmacy, University of Chicago Medicine, Chicago, Illinois, USA; Infectious Diseases and Critical Care Medicine, Carle Health Methodist Hospital, Peoria, Illinois, USA; Section of Cardiac Surgery, Department of Surgery, University of Chicago Medicine, Chicago, Illinois, USA; Section of Infectious Diseases and Global Health, University of Chicago Medicine, Chicago, Illinois, USA

**Keywords:** fungal endocarditis, *Scopulariopsis*, *Scopulariopsis* endocarditis, multi-drug resistant fungi, intraoperative TEE and video

## Abstract

We present the first case of native aortic valve endocarditis caused by *Scopulariopsis.* Intraoperative images and videos from valve replacement surgery illustrate the severity of fungal endocarditis. This case demonstrates the aggressive presentation of left-sided fungal endocarditis, highlights challenges with treating highly resistant fungi, and considers the potential utility of olorofim.

## Teaser

This report describes the diagnosis and management of native aortic valve endocarditis caused by Scopulariopsis species. Intraoperative echocardiograms and videos highlight the large vegetations associated with fungal endocarditis. The case further discusses the challenges of treating infections caused by resistant molds.

## CASE REPORT

A 47-year-old woman with rheumatic mitral valve disease underwent surgery for mechanical mitral valve replacement (day 0) and was discharged on day 5. She returned to the hospital on day 9 with an arterial pseudoaneurysm at the access site from the recent procedure and underwent open repair of the pseudoaneurysm on day 10. She was discharged with a plan to allow the surgical site to heal by secondary intention. During the ensuing months, she was intermittently treated with antibiotics for recurrent presumed bacterial cellulitis at the surgical site, with relief of symptoms after completing each course of antibiotics.

**Figure 1.**
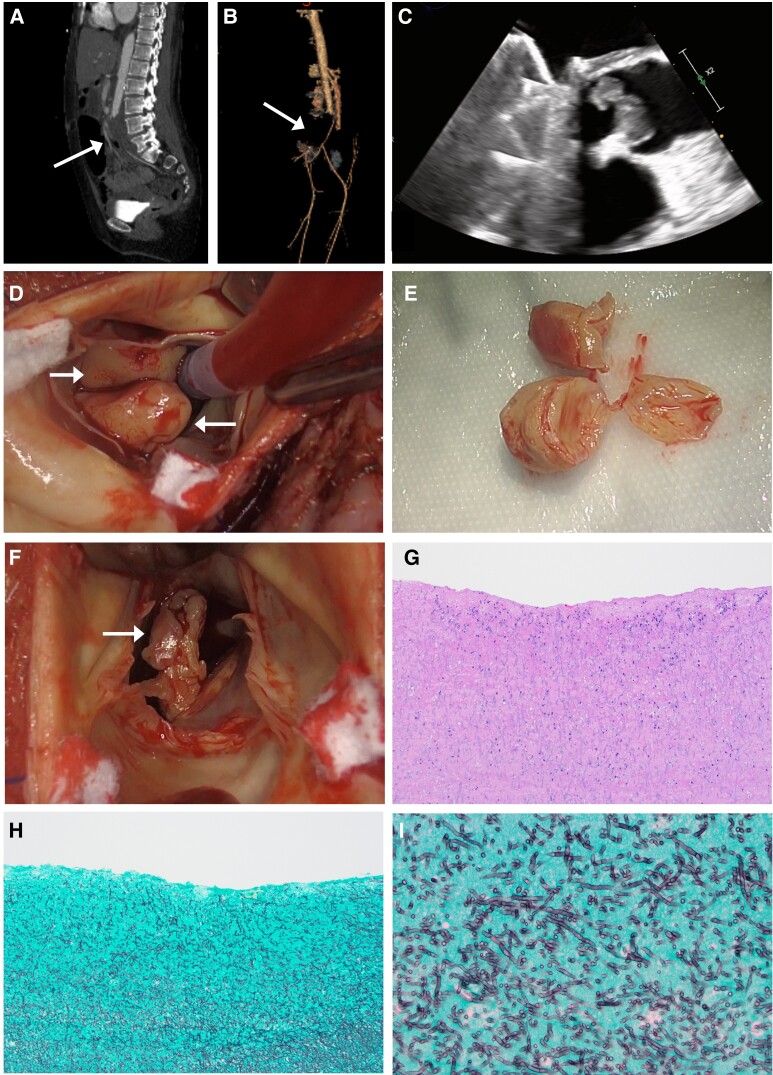
Representative images from this case. *A* and *B*, Computed tomography angiogram and 3-dimensional reconstruction reveal near-complete occlusive thrombus (arrows) at the aortic bifurcation, with only a sliver of a residual perfusion seen to the right common femoral artery. The thrombus was later retrieved surgically (day 136), and cultures revealed *Scopulariopsis*. *C–I*, Representative images from cardiac valve replacement surgery on day 151. *C*, Intraoperative transesophageal echocardiogram demonstrates large vegetations adherent to the native aortic valve. *D*, Intraoperative view of multiple large circumscribed vegetations (arrows) on the native aortic valve. *E*, Explanted native aortic valve with adherent vegetations. *F*, Intraoperative view of an irregular friable vegetation (arrow) on the mechanical mitral valve. *G*, Hematoxylin and eosin stain (10× magnification) of cardiac valve vegetation. *H* and *I*, Grocott methenamine silver staining at 10× and 40× magnification of the cardiac valve vegetation displays abundant septate hyphae with branching at acute angles, with cultures later confirming *Scopulariopsis* species by day 165.

On day 92, she developed acute weakness and numbness of bilateral legs, and computed tomography angiography revealed a large occlusion of the distal abdominal aorta with extension into the common iliac arteries (Figure 1*A* and 1*B*). Transesophageal echocardiogram demonstrated a large pedunculated mass originating from the mechanical mitral valve and extending into the left ventricular outflow tract. Cardiac surgery was considered, although a repeat transesophageal echocardiogram conducted on day 102 for surgical planning detected no vegetation or thrombus; thus, surgery was deferred. She was afebrile, blood cultures remained without growth, and workup for culture-negative endocarditis was unremarkable.

On day 136, she underwent elective aortoiliac bypass graft surgery to manage the distal abdominal aorta occlusion. A 6.8 × 4.0 × 1.5–cm aortic thrombus was retrieved, and pathology demonstrated abundant septate hyphae with branching at acute angles, which was reminiscent of *Aspergillus* species and prompted the initiation of voriconazole. Cultures later confirmed *Scopulariopsis* species, and she was subsequently transitioned to empirical liposomal amphotericin B and micafungin, pending susceptibilities. On day 138, she developed acute left-sided weakness and left facial droop, and magnetic resonance imaging brain disclosed multifocal infarcts. A transthoracic echocardiogram identified a 1.4-cm mobile echodensity attached to the aortic valve.

She was transferred to a tertiary care hospital on day 149 for advanced management. Admission laboratory results were notable for a total leukocyte count of 22.7 × 10^3^/µL (reference range, 3.5–11 × 10^3^/µL) but were otherwise unremarkable. A transthoracic echocardiogram redemonstrated a large vegetation in the left ventricular outflow tract, and computed tomography angiography noted a retroperitoneal hematoma measuring 16.5 × 9.4 × 5.3 cm. Surgical management of endocarditis was urgent; however, the case was complicated by the retention of prosthetic aortic graft material from the bypass surgery on day 136 and the large retroperitoneal hematoma, both of which were likely seeded by *Scopulariopsis*. Nevertheless, to limit the risk of further morbidity from endocarditis, she underwent cardiac valve replacement surgery on day 151, with replacement of the aortic and mitral valves with bioprosthetic valves (Figure 1*CitalicF*, Supplementary Videos 1 and 2). Cultures from the vegetations identified *Scopulariopsis* species (Figure 1*GitalicI*). She was treated with voriconazole, micafungin, and terbinafine, which were later narrowed to micafungin and terbinafine based on susceptibilities (Table 1, day 151 culture).

**Table 1.** Antifungal Susceptibility Testing Data Indicating Minimum Inhibitory Concentration

		Day 151: Cardiac Valve Vegetation	
Drug	Day 136: Aortic Thrombus (ARUP)	UTSA	ARUP
Amphotericin B	≥8	0.5	4
Anidulafungin	0.5	…	1
Caspofungin	0.12	…	0.5
Ibrexafungerp	…	8	…
Isavuconazole	…	4	…
Itraconazole	≥16	…	≥16
Micafungin	0.12	0.125	0.5
Olorofim	…	0.03	…
Posaconazole	≥16	1	≥16
Terbinafine	…	1	…
Voriconazole	≥16	4	8

Antifungal susceptibility testing results are displayed. *Scopulariopsis* cultured from the thrombus retrieved from the abdominal aorta during surgery on day 136 underwent antifungal susceptibility testing via broth microdilution at ARUP Labs, Salt Lake City, Utah, with results available by day 162. Similarly, *Scopulariopsis* cultured from the cardiac valve vegetations excised during surgery on day 151 were sent for susceptibility testing to the University of Texas San Antonio (UTSA), San Antonio, Texas, where testing was also performed via broth microdilution, with susceptibility results available by day 213. As there was some discordance between these results, cultures from the cardiac valve vegetation from surgery on day 151 were also sent to ARUP Labs for susceptibility testing.

The immediate postoperative course was complicated by hemodynamically unstable supraventricular tachycardia, followed by cardioversion and subsequent acute right ventricle failure necessitating cannulation to oxyRVAD (oxygenated right ventricular assist device). She was successfully decannulated from the oxyRVAD on day 164. The course was also notable for oliguric kidney injury requiring dialysis and persistent left hemothorax requiring decortication on day 176.

Unfortunately, a transthoracic echocardiogram on day 180 revealed a new mobile echodensity on the prosthetic aortic valve, concerning for recurrent endocarditis. She continued taking micafungin and terbinafine for suppression as she was not a candidate for repeat surgery. By day 191, she developed acute embolic strokes, later complicated by hemorrhagic conversion and devastating neurologic injury, and she transitioned to comfort measures on day 194.

## DISCUSSION

Here, we present a case of prosthetic mitral valve and native aortic valve endocarditis caused by *Scopulariopsis* species. The patient successfully underwent replacement of the cardiac valves; however, she developed recurrent prosthetic valve endocarditis and embolic disease and ultimately died. This case illustrates the morbidity of fungal endocarditis, highlights the important medical and surgical considerations for the management of these complicated cases, and underscores the urgent need to develop antifungals to target multidrug-resistant fungi.

### Epidemiology of Fungal Endocarditis

Fungal endocarditis represents 1% to 3% of all cases of endocarditis, with >50% of cases caused by *Candida* species and with molds constituting a minority of cases [1]. Risk factors include immunosuppression, prolonged antibiotic use, prior cardiac surgery, the presence of implantable cardiac devices, and injection drug use [1, 2]. Diagnosis of fungal endocarditis is challenging, as symptoms are frequently milder than what is typically observed in bacterial endocarditis; patients with fungal endocarditis may present with smoldering symptoms, such as weight loss, diaphoresis, and malaise [2]. Importantly, fungal endocarditis is often associated with large vegetations (>1 cm), and the emergence of embolic phenomena—particularly stroke, pulmonary embolism, acute limb ischemia, and endogenous endophthalmitis—may be the first manifestations [1, 2]. Furthermore, identification of the causative organism and initiation of appropriate therapy are often delayed by slow-growing cultures, and fungal markers, such as β-D-glucan and galactomannan, are sometimes employed to guide empiric therapy [2]. Management with early surgical debridement is critical, and valve replacement is often required to prevent embolic disease [3]. Antifungal therapy is typically administered for at least 6 weeks, although lifelong suppressive antifungals are often considered [2]. Nevertheless, fungal endocarditis has a mortality exceeding 70%, which is attributed to host factors, delays in diagnosis, difficulties with source control, and inadequate therapies [1].

### Review of *Scopulariopsis* Endocarditis

*Scopulariopsis* is a ubiquitous environmental fungus. Infections are typically confined to localized processes, such as onychomycosis and keratitis. *Scopulariopsis* rarely causes invasive infections, with manifestations including deep cutaneous infection, brain abscess formation, sinopulmonary infection, and endocarditis [4]. *S brevicaulis* is most commonly identified in severe infections [4].

Nine cases of endocarditis caused by *Scopulariopsis* are documented in the literature and are summarized in Table 2 [5–13]. Eight cases identified vegetations associated with prosthetic cardiac valves. An additional case was reported in association with a pacemaker lead vegetation that extended to abut the native tricuspid valve [12]. However, none of these prior cases documented the formation of vegetations on native cardiac valves, as we describe here. All prior cases received surgical debridement, and most cases received antifungal therapy guided by susceptibilities. Nonetheless, there was significant morbidity and mortality with endocarditis, as in the present case.

**Table 2.** Review of Prior Cases of Endocarditis Caused by *Scopulariopsis* Species

Case	Age, y; Sex	Valves Affected	Relevant Medical History	Metastatic Foci	Surgical Debridement	Main Medical Therapy	Treatment Success?
Arroyo [13]	37; F	Prosthetic aortic valve	Repaired congenital aortic aneurysm	Left iliac artery thrombus	Yes	Posaconazole	Yes
Cawcutt [8]	65; M	Prosthetic mitral valve	Mitral regurgitation	Left popliteal aneurysm and thrombus, bilateral renal infarcts	Yes	Caspofungin, voriconazole, posaconazole	Yes
Chen-Scarabelli [9]	38; F	Prosthetic mitral valve	Mitral rheumatic disease	Multifocal strokes	Yes	Caspofungin	Yes
Gentry [10]	36; M	Prosthetic mitral valve	Mitral rheumatic disease	Bilateral renal infarcts	Yes	Itraconazole	Yes
Guo-tao [12]	49; M	Pacemaker-associated native tricuspid valve	Bradycardia	None identified	Yes	Voriconazole	Yes
Isidro [5]	67; F	Prosthetic mitral valve	Mitral rheumatic disease, pulmonary hypertension, atrial fibrillation, congestive heart failure, prosthetic aortic valve	Right Iliac and femoral artery thrombi	Yes	Amphotericin, voriconazole	Yes: death reported to be unrelated
Jain [6]	58; M	Prosthetic mitral valve	Coronary artery disease, repaired ischemic mitral regurgitation	None Identified	Yes	Voriconazole	Yes
Migrino [7]	67; M	Prosthetic aortic valve	Repaired aortic stenosis, onychomycosis	Right profunda femoris, left common femoral artery thrombi	Yes, twice	Amphotericin, fluconazole	No: acute myocardial infarction, vegetation obstructing right coronary artery ostium
Muehrcke [11]	Not provided	Prosthetic aortic valve	Onychomycosis, unknown aortic valve pathology	Recurrent lower extremity emboli	Yes	Amphotericin, fluconazole (panresistant specimen)	No: uncontrolled fungal sepsis

### Approaches to Treatment

There are limited options to treat invasive infections caused by *Scopulariopsis*, and antifungal susceptibility testing often reveals high minimum inhibitory concentrations (MICs) for antifungals when tested against clinical isolates of *Scopulariopsis* [14, 15]. Notably, there are no established breakpoints because there are limited data with which to correlate MICs to clinical outcomes; thus, antifungal selection is often driven by clinical responses to therapy [14]. Localized infections such as onychomycosis have been successfully eradicated with monotherapy with terbinafine or triazoles; however, combination therapy is the recommended approach to invasive infections, with current guidelines recommending a triazole such as voriconazole or isavuconazole as first-line therapy, followed by liposomal amphotericin B plus voriconazole as an alternative regimen and a combination of a triazole, micafungin, and terbinafine as a salvage regimen [14, 16–20].

In the present case, the patient started taking empiric voriconazole and liposomal amphotericin B due to an initial concern for *Aspergillus* based on the preliminary pathology from the day 136 surgical procedure (Figure 1*GitalicI*). Once *Scopulariopsis* was confirmed by culture, liposomal amphotericin B was discontinued due to reports of high MICs in the literature and the possibility of intrinsic resistance among *Scopulariopsis* species [14]. Following the cardiac valve replacement procedures on day 151, she was treated with micafungin and terbinafine, with a plan to continue these agents indefinitely.

Importantly, the antifungal susceptibility data presented in Table 1 indicate divergent susceptibility patterns between the cultures yielded from the day 136 aortic thrombus and the day 151 cardiac vegetations, despite both these cultures detecting *Scopulariopsis* species. Susceptibility testing was performed at ARUP Labs, Salt Lake City, Utah, and the University of Texas San Antonio, San Antonio, Texas, respectively. These reference laboratories utilize broth microdilution for antifungal susceptibility testing. Therefore, to reconcile these differences, the day 151 culture was also sent to ARUP Labs, with the results shown in Table 1 indicating generally concordant MICs between the laboratories (within 1 dilution). However, the susceptibility patterns to posaconazole and amphotericin B were quite dissimilar. These differences may be attributed several factors, including variability in testing between laboratories and the challenges of antifungal susceptibility testing in vitro. We also considered the possibility of different circulating isolates of *Scopulariopsis* in the patient, particularly as a consequence of selective pressure from antifungal treatment exerted between the cultures; yet, whole genome sequencing to probe this question is beyond the scope of this case.

Our patient developed recurrent endocarditis within 1 month of valve replacement despite antifungal therapy. We suspect that the retained vascular graft was likely seeded and acted as a persistent source of *Scopulariopsis* that provoked recurrent endocarditis despite appropriate therapy. Furthermore, the replaced cardiac valves were not expected to have endothelialized within 1 month after valve surgery and were particularly vulnerable to reseeding as a result.

Prior reports note that chronic antifungal suppressive therapy after valve replacement surgery may have a marginal impact on preventing recurrent fungal endocarditis [1, 11]. Therefore, in addition to ensuring aggressive source control, it is necessary to consider properties of *Scopulariopsis* and other highly resistant molds that render them so challenging to treat. Specific mechanisms of antifungal resistance have not yet been elucidated for *Scopulariopsis*. However, we speculate that *Scopulariopsis* may harbor similar mechanisms of antifungal resistance to those employed by other multidrug resistant molds, such as *Scedosporium* and *Lomentospora*.

Specifically, *Scedosporium* and *Lomentospora* express efflux pumps and form biofilms, both of which contribute to their ability to withstand diverse classes of antifungals [21]. Biofilms are a complex ecosystem where extracellular matrix proteins produce a network that regulates access of a microbial community to oxygen, metabolites, and circulating proteins [22]. A recent study evaluated antifungal susceptibilities of *Scedosporium* and *Lomentospora* when assessed in their planktonic state as compared with their biofilm state [21]. Biofilms of *Scedosporium* and *Lomentospora* demonstrated significantly increased activity of efflux pumps and the production of extracellular matrix components and antioxidative stress responses, with these strategies collectively contributing to 2- to 16-fold increases in the MICs to triazoles [21]. This has important clinical implications, as biofilm production is often observed in unremitting infections by bacteria and fungi.

Accordingly, antimicrobial medications that impair the production and maintenance of biofilms have the potential to significantly affect these challenging infections. Olorofim is an antifungal medication currently under development and acts to inhibit de novo fungal pyrimidine biosynthesis [23, 24]. Pyrimidine biosynthesis, which is essential for DNA replication, has also been identified as a critical mediator of biofilm formation for many clinically relevant bacterial pathogens and may have a similar role in molds [25–27]. Encouragingly, olorofim exerts potent antifungal activity against many highly resistant molds in vitro, including *Scopulariopsis*, *Scedosporium*, and *Lomentospora*, and it maintains robust antifungal activity against *Lomentospora* biofilms in vitro [24, 28]. It remains unclear if the mechanism of antifungal activity of olorofim is related to its ability to inhibit biofilms, although this may be a conserved pathway that could be exploited for the development of new therapeutics.

In this case, the patient's *Scopulariopsis* culture demonstrated a MIC of 0.03 µg/mL to olorofim. While there are no established breakpoints with which to interpret this MIC, the MIC of olorofim in vitro ranged from 0.008 to >2 µg/mL with a mode of 0.06 µg/mL in a recent survey against isolates of *Scedosporium* and *Lomentospora* [29]. Unfortunately, olorofim was not available for this patient. However, given the patient's recurrent endocarditis and retained prosthetic material, achieving remission of the infection with antifungals would have been challenging without complete source control.

In summary, fungal endocarditis is a highly morbid disease, and effective management requires urgent valve replacement surgery, aggressive debridement of all foci of infection, and the prolonged use of a combination antifungal therapy, including agents that suppress biofilm formation.

## Supplementary Material

ofae323_Supplementary_Data

## Supplementary Data

Supplementary materials are available at *Open Forum Infectious Diseases* online. Consisting of data provided by the authors to benefit the reader, the posted materials are not copyedited and are the sole responsibility of the authors, so questions or comments should be addressed to the corresponding author.

## Notes

***Acknowledgments.*** We acknowledge Daniel Montelongo, PhD, Stephen Weber, MD, and Nathan Wiederhold, PharmD, for their advice and support.

***Patient consent.*** The patient's written consent was obtained, and images presented here were anonymized.
